# Editorial: Advances in Mechanisms of Renal Fibrosis

**DOI:** 10.3389/fphys.2018.00284

**Published:** 2018-03-23

**Authors:** Hui Y. Lan, David J. Nikolic-Paterson

**Affiliations:** ^1^Department of Medicine and Therapeutics, Li Ka Shing Institute of Health Sciences, Chinese University of Hong Kong, Hong Kong, China; ^2^Department of Nephrology, Monash Medical Centre, Monash University Centre for Inflammatory Diseases, Monash Health, Clayton, VIC, Australia

**Keywords:** BMP7, fibroblast, HIPK2, JNK, miRNA, Smad, TGF-beta

Scarring of the glomerular and tubulointerstitial compartments is a hallmark of progressive kidney disease and is considered a common pathway leading to end-stage of renal failure. Renal fibrosis involves a complex interplay between intrinsic kidney cells, leukocytes, and fibroblasts in which transforming growth factor-β (TGF-β) plays a key role. Inhibition of TGF-β1 suppresses renal fibrosis in a number of animal models; however, TGF-β1 is also a negative regulator of the immune response so that targeting this key factor has been a difficult proposition. Much effort has focused on how TGF-β1 promotes renal fibrosis to identify other steps that can be targeted safely. The articles in this eBook describe advances in our understanding in the mechanisms of renal fibrosis that operate upstream and downstream of TGF-β/Smad signaling.

TGF-β signals via canonical (Smad-based) and non-canonical (non-Smad based) pathways (Meng et al.). An important development is the demonstration that Smad2 and Smad3 exert opposing roles in renal fibrosis, with Smad2 being anti-fibrotic and Smad3 pro-fibrotic. This provides potential avenues for manipulating the Smad2/3 balance within Smad2/3/4 complexes to alter the outcome of TGF-β/Smad signaling. Two other proteins can suppress canonical TGF-β/Smad signaling. First, Smad7 has a negative feed-back role in TGF-β/Smad signaling. Genetic strategies to over-express Smad7 in mice inhibits renal fibrosis with the challenge being how to translate these proof of principle studies into the clinic (Meng et al.). Second, bone morphogenetic protein 7 (BMP7) is a member of the TGF-β super family which exerts anti-fibrotic effects in many models of renal fibrosis (Li et al.). This is attributed to counterbalancing the pro-fibrotic effects of TGF-β such as reducing collagen formation, increasing matrix degradation and inactivating matrix-producing cells. However, the promise of recombinant BMP7 as an anti-fibrotic therapy has yet to be established in the clinic (Li et al.).

## Mechanisms upstream of TGF-β signaling

Several mechanisms are described in this eBook which operate upstream of TGF-β/Smad signaling. Homeodomain-interacting-protein kinase 2 (HIPK2) was identified as an important regulator of inflammation and fibrosis in a mouse model of HIV-associated nephropathy acting upstream of both NF-kB and TGF-β signaling (Nugent et al.). *Hipk2* deficient mice are viable and show protection against renal fibrosis in animal models. However, the clinical use of HIPK2 inhibitors may be problematic since HIPK2 also functions as a tumor suppressor and loss of HIPK2 may lead to neurodegenerative disease.

A mainstay of therapy for chronic kidney disease is inhibition of the production or action of angiotensin II (Ang II). Indeed, Ang II is an inducer of TGF-β production and activation. Further opportunities for targeting renal fibrosis are evident in the non-classical renin-angiotensin system (RAS), including the ACE2/Ang(1–7)/Mas receptor axis, the (pro)renin receptor, and the Ang A/alamandine/MrgD axis (Lv and Liu). The challenge is to understanding the precise role of these non-classical members of the RAS in renal fibrosis and in selecting the most effective therapeutic strategies.

Stress-induced activation of the c-Jun amino terminal kinase (JNK) pathway in cells of the glomerular and tubulointerstitial compartments is a common feature of chronic kidney disease (Grynberg et al.). Pharmacologic inhibition of JNK suppresses inflammation, fibrosis, and apoptosis in several models of renal fibrosis. JNK signaling acts to increase TGF-β1 expression, to promote activation of latent TGF-β1, and to promote transcription of pro-fibrotic molecules via direct phosphorylation of the linker region of Smad3. However, a lack of efficacy of JNK inhibitors in models of diabetic nephropathy and the recent failure of JNK inhibition in a trial of idiopathic pulmonary fibrosis have raised questions regarding how best to target this pro-fibrotic mechanism (Grynberg et al.).

Histone deacetylases (HDAC) are a group of enzymes that induce deacetylation of both histone and non-histone proteins and thereby modify many cellular functions. Pan- or class-specific HDAC inhibitors can suppress the activation and proliferation of cultured renal fibroblasts and attenuate renal fibrosis in animal models (Liu and Zhuang). While clinical trials of HDAC inhibitors are progressing in cancer, this has yet to be reported for kidney disease. A major challenge is whether chronic inhibition of one or more HDAC enzymes can suppress renal fibrosis without significant side-effects of enzyme inhibition.

## Mechanisms downstream of TGF-β signaling

Substantial progress has been made in identifying miRNA molecules which regulate TGF-β/Smad3 induced fibrosis. In particular, TGF-β promotes fibrosis by increasing levels of miR-21, miR-433, and miR-192 which amplify TGF-β signaling and promote de-differentiation of tubular epithelial cells (Chung and Lan). In addition, TGF-β signaling reduces levels of the miR-29 and miR-200 families which protect against renal fibrosis. The delivery of modified oligonucleotides or plasmid-based expression constructs to up- or down-regulate miRNA levels in tissues is an active area of clinical research, particularly in cancer, although many issues will need to be addressed before chronic administration of such reagents can be performed in fibrotic kidney disease (Chung and Lan).

Studies by Yan et al. have described the recruitment of bone marrow-derived fibroblasts in models of tubulointerstitial fibrosis. In response to injury, tubular epithelial cells release chemokines (CCL21/CXCL16/CCL2) and TGF-β1. These chemokines recruit monocytes and fibrocytes from the circulation while TGF-β1 plus other factors, such as adiponectin and Jak3/STAT6 signaling, induce transition of these cells into collagen producing myofibroblasts thereby promoting renal fibrosis (Yan et al.). These findings identify several potential therapeutic targets to inhibit the recruitment and activation of bone marrow cells in the injured kidney.

Finally, an exciting new finding based on careful lineage tracing studies is the identification of a population of renal erythyropoietin-producing (REP) cells as a significant source of collagen producing interstitial myofibroblasts (Souma et al.). Hypoxia activates fibroblasts in culture and hypoxia is a common feature in renal fibrosis. REP cells represent a direct mechanism by which hypoxia induces myofibroblast transition of intrinsic renal fibroblast-like cells. What is particularly interesting is the finding that such transformed REP cells can recover their original physiological properties upon resolution of hypoxia.

In conclusion, these articles provide a detailed description of both the key TGF-β/Smad signaling pathway as well as other mechanisms involved in renal fibrosis both upstream and downstream of TGF-β. The wide variety of potential new targets described herein bodes well for the future development of effective therapies to tackle the major clinical problem of progressive renal fibrosis.

## Author contributions

DN-P and HL reviewed all the papers included in the Research Topic of Frontiers in Renal and Epithelial Physiology and summarized in the Editorial their main findings, together with a commentary on the current knowledge about renal fibrosis.

### Conflict of interest statement

The authors declare that the research was conducted in the absence of any commercial or financial relationships that could be construed as a potential conflict of interest.

